# Transgenerational Tolerance to Salt and Osmotic Stresses Induced by Plant Virus Infection

**DOI:** 10.3390/ijms232012497

**Published:** 2022-10-18

**Authors:** Francisco J. Hernández-Walias, Marina García, Marina Moreno, Ioannis Giannoukos, Natalia González, Eugenio Sanz-García, Khouloud Necira, Tomás Canto, Francisco Tenllado

**Affiliations:** 1 Department of Microbial and Plant Biotechnology, Margarita Salas Center for Biological Research, CSIC, Ramiro de Maeztu 9, 28040 Madrid, Spain; 2 Laboratory of Molecular Genetics, Immunology and Biotechnology, Faculty of Sciences, University of Tunis El Manar, Manar II, Tunis 2092, Tunisia

**Keywords:** *Potato virus X*, priming response, plant defense, abiotic stress, reproductive fitness

## Abstract

Following pathogen infection, plants have developed diverse mechanisms that direct their immune systems towards more robust induction of defense responses against recurrent environmental stresses. The induced resistances could be inherited by the progenies, rendering them more tolerant to stressful events. Although within-generational induction of tolerance to abiotic stress is a well-documented phenomenon in virus-infected plants, the transgenerational inheritance of tolerance to abiotic stresses in their progenies has not been explored. Here, we show that infection of *Nicotiana benthamiana* plants by *Potato virus X* (PVX) and by a chimeric *Plum pox virus* (PPV) expressing the P25 pathogenicity protein of PVX (PPV-P25), but not by PPV, conferred tolerance to both salt and osmotic stresses to the progeny, which correlated with the level of virulence of the pathogen. This transgenerational tolerance to abiotic stresses in the progeny was partially sustained even if the plants experience a virus-free generation. Moreover, progenies from a *Dicer-like3* mutant mimicked the enhanced tolerance to abiotic stress observed in progenies of PVX-infected wild-type plants. This phenotype was shown irrespective of whether *Dicer-like3* parents were infected, suggesting the involvement of 24-nt small interfering RNAs in the transgenerational tolerance to abiotic stress induced by virus infection. RNAseq analysis supported the upregulation of genes related to protein folding and response to stress in the progeny of PVX-infected plants. From an environmental point of view, the significance of virus-induced transgenerational tolerance to abiotic stress could be questionable, as its induction was offset by major reproductive costs arising from a detrimental effect on seed production.

## 1. Introduction

Under field conditions, plants are exposed to a number of biotic and abiotic stresses, including fungal, bacterial and viral infections, as well as situations of drought and salinity, among others [1]. Each of these stresses induces physiological, biochemical and molecular changes, which can negatively impact plant growth and productivity [2]. Plants often respond to biotic and abiotic stresses by inducing stress resistance, a network of signals and responses occurring at the molecular, cellular and physiological levels that lead to an effective activation of defense mechanisms. [3]. Induced resistance is generally associated with a physiological state, known as immunity priming, in which plants can react more efficiently to recurrent environmental stresses [4]. Most examples of the priming responses tend to protect plants against pathogens with lifestyles similar to the one causing the response [5]. While salicylic acid (SA) plays a key role in defense responses against biotrophic pathogens, jasmonic acid (JA) is considered the main hormonal route implicated in resistance to necrotrophic ones and herbivores [6].

Although priming responses have mostly been studied within the lifespan of individuals, there is increasing evidence that plants have a memory of encountered stress situations that can be transmitted to their progenies. For example, progeny of *Arabidopsis thaliana* plants that had been either treated with β-aminobutyric acid (BABA) or inoculated with an avirulent isolate of the bacteria *Pseudomonas syringae* pv tomato showed enhanced resistance to virulent *P. syringae* pv tomato and the oomycete *Hyaloperonospora arabidopsidis* [7,8]. Enhanced virus resistance was reported in the progeny of tobacco (*Nicotiana tabacum*) plants infected with *Tobacco mosaic virus* (TMV) [9]. Furthermore, Rasmann et al. [10] showed that Arabidopsis and tomato (*Solanum lycopersicum*) submitted to herbivory or mechanical damage produce offspring that were more resistant against herbivores. With regard to abiotic stress, exposure to high temperatures led to enhanced heat tolerance in the following generations in Arabidopsis [11], and the progeny of plants exposed to salt exhibited higher tolerance in the subsequent generation [12]. Epigenetic processes, which include DNA methylation and histone modifications, are a likely mechanism to keep stress-response memory in following generations [7,13]. Small interfering RNAs (siRNAs), approximately 24-nucleotide (nt) RNAs that are processed from double-stranded RNA by Dicer-like3 (DCL3), can lead to epigenetic regulation in a process known as RNA-directed DNA-methylation (RdDM) [14]. It has been proposed that transgenerational resistance can be transmitted by hypomethylated genes that direct priming of defenses in the following generations [7]. Some of these transgenerational effects can persist over multiple generations and have the potential to provide adaptive benefits to progenies, thereby enhancing the evolutionary fitness of the parents [5,15].

Plant viruses are biotrophic pathogens that require living tissues for their multiplication and dispersion [16]. To accomplish their life cycle, viruses must interfere with the functions of different host metabolic pathways, altering the plant physiology and cellular homeostasis. Most often, such metabolic shifts caused by virus infections lead to a decrease in plant fecundity that provokes a detrimental effect on the overall host fitness [17]. Because virulence, defined as the deleterious effect of a pathogen on host fitness, does not represent any adaptive advantage for obligate pathogens, it is not obvious why viruses damage their hosts. However, several reports have hinted that mechanisms and processes operating in compatible interactions between plants and viruses might offer plants a better performance under abiotic stresses. For instance, it has been shown that virus infection can enhance the resilience of host plants to cold and drought [18,19]. In addition, infection of Arabidopsis by different viruses rendered seeds with improved tolerance to deterioration by elevated temperature [20]. It is worth mentioning that other forms of compensatory effects by viruses to their hosts have been reported. For instance, it has been shown than plants of several species were protected from herbivory by virus infection [21] and that virus infection attracts pollinating insects in tomato [22].

Viral-encoded proteins are usually elicitors of defense responses in plants. Indeed, several works have linked trade-offs in compatible interactions between plants and viruses to a group of viral proteins commonly known as pathogenicity determinants. For instance, the 2b protein of *Cucumber mosaic virus* has been shown to be involved in both tolerance to drought in Arabidopsis [23] and insect pollinator attraction in tomato [22]. The transgenic expression of the helper component-proteinase protein from *Tobacco etch virus* in tobacco plants conferred a resistant phenotype to TMV and to the oomycete *Peronospora tabacina* [24]. Similarly, infection by *Plum pox virus* (PPV) expressing the P25 protein of *Potato virus X* (PVX) conferred an enhanced drought-resistant phenotype to *Nicotiana benthamiana* and Arabidopsis plants compared to infections with either PPV or PVX [18]. The P25 protein is multifunctional, participating in viral movement and acting as a suppressor of RNA silencing. Furthermore, several pathogenicity determinants of plant viruses have been reported to induce repression of DNA methylation in their hosts [25]. Although within-generational induction of tolerance to abiotic/biotic stress is a well-documented phenomenon in virus-infected plants, the transgenerational inheritance of tolerance to abiotic stresses, particularly those exacerbated by global warming, to progenies has not been explored. No PVX or PPV isolate has been identified as transmitted by seed in *N. benthamiana*, so vertical transmission of these viruses from infected mother plants to their progeny does not occur [26]. The aim of this work was to investigate whether plants infected with viruses displaying different levels of virulence produce progenies tolerant to abiotic stress. We utilized PVX and PPV, members of the genera *Potexvirus* and *Potyvirus*, respectively, and *N. benthamiana*, a model plant adopted by virologists due to its general susceptibility to virus infection. We found that when exposed to salt or osmotic stresses, the progeny of PVX-infected plants showed enhanced tolerance to stress compared to the progeny of non-treated plants, which correlated with an enhanced expression of stress-related genes. A *N. benthamiana* mutant (Dcl3) that was deficient in the biogenesis of 24-nt siRNAs exhibited constitutively increased tolerance to abiotic stress. We further provide evidence that these transgenerational effects are sustained even after one virus-free generation. However, they come at the expense of reductions in reproductive fitness.

## 2. Results

### 2.1. Seed Tolerance to Abiotic Stresses Is Affected Differentially by Virus Infection of the Parental Plants

**Figure 1:**
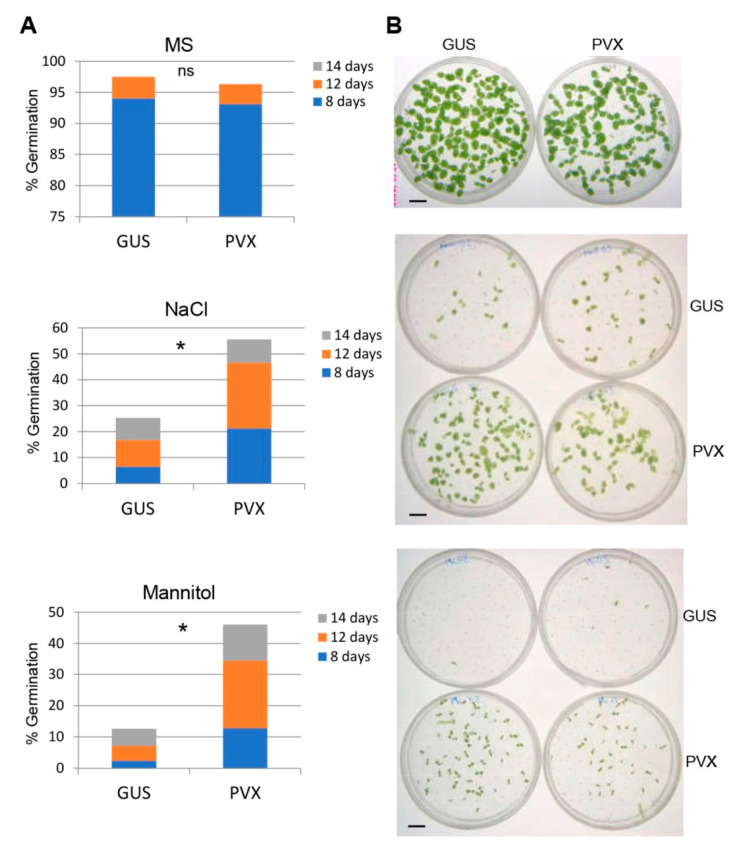
Tolerance to abiotic stress in the progeny of PVX-infected *N. benthamiana* plants: (**A**) germination rates of progenies derived from mock-inoculated plants (Gus) and plants infected with PVX on MS medium alone (upper panel) or supplemented with either 100 mM NaCl (middle panel) or 200 mM mannitol (lower panel); (**B**) representative examples of the different treatments photographed at 14 days after sowing. The accumulative germination rates were calculated at 8, 12 and 14 days after sowing. Values were obtained from two independent experiments using six progenies per treatment. The significant differences are indicated by asterisk (Student’s t-test, * *p* < 0.05); ns, not significative; bars in each panel represent 1 cm.

To investigate whether virus infection affects tolerance to abiotic stresses transgenerationally, we collected seeds from *N. benthamiana* plants that had been mock-inoculated (Gus) or infected with PVX. The germination capacity of seeds derived from mock-inoculated plants and plants infected with PVX was evaluated on Murashige and Skoog (MS) medium alone or supplemented with either NaCl (100 mM) or mannitol (200 mM). Previously, we confirmed by Western blot analysis that seedlings derived from PVX-infected plants were not infected by virus. There were no significant differences in the germination rates of the progenies derived from mock-inoculated and PVX-infected plants under control conditions (Figure 1). However, the germination of both progenies was inhibited by mannitol and NaCl although at a different level. Remarkably, progenies from PVX-infected plants were more tolerant to both abiotic stresses than mock-inoculated ones, resulting in germination rates of progenies from PVX-infected plants being approximately 2.2- and 3.6-fold higher than that of mock-inoculated plants in the presence of NaCl and mannitol, respectively, 14 days after sowing.

**Figure 2:**
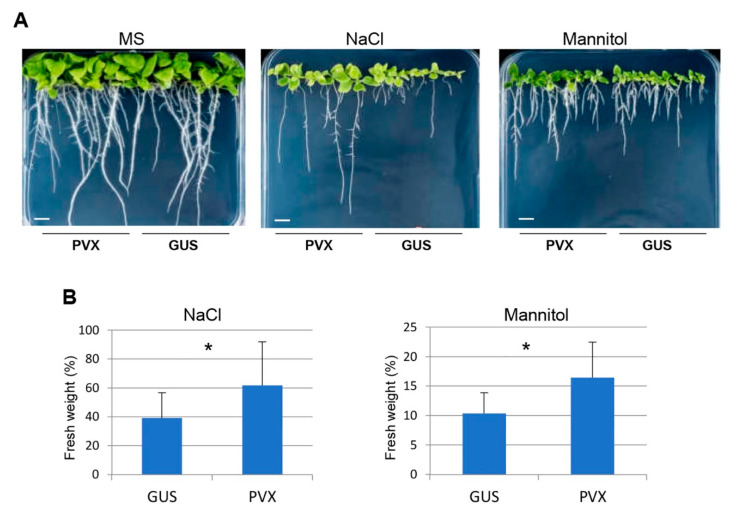
Tolerance to abiotic stress on the growth of progeny seedlings derived from PVX-infected *N. benthamiana* plants: (**A**) phenotype of seedlings derived from mock-inoculated plants (Gus) and plants infected with PVX germinated on MS medium for 7 days and then transferred to MS medium alone (left) or supplemented with either 150 mM NaCl (middle) or 200 mM mannitol (right); (**B**) relative fresh weight of seedlings shown in (**A**) at 13 days. Values are represented relative to the corresponding values of seedlings grown on MS medium. Mean values and SD were obtained from two independent experiments using two progenies per treatment (n = 16). The significant differences are indicated by asterisk (Student’s t-test, * *p* < 0.05). Bars in each panel represent 1 cm.

Next, we tested whether virus infection played a role in seedling establishment in the progeny of PVX-infected plants under stress conditions. *N. benthamiana* seedlings germinated on MS medium were transferred to vertical growth plates containing MS medium alone or supplemented with either NaCl (150 mM) or mannitol (200 mM) 7 days after sowing. Under stress conditions, the seedlings derived from mock-inoculated plants showed enhanced root growth inhibition compared with those derived from PVX-infected plants (Figure 2A). Moreover, the relative fresh weights of PVX-derived progenies were increased by 1.6- and 1.5-fold, compared with those of mock-inoculated progenies in the presence of NaCl and mannitol, respectively (Figure 2B).

Several viruses, including PVX, may cause an increase in the accumulation of SA, a plant hormone implicated in tolerance to abiotic stress [27]. To determine whether SA contributes to abiotic stress tolerance in the progeny of PVX-infected plants, seeds were collected in parallel from *N. benthamiana* transgenic plants expressing the SA-degrading enzyme salicylate hydroxylase (NahG) and from the wild-type (Wt) control, which were either infected with PVX or mock-inoculated. Seeds derived from six independent progenies per treatment were germinated on MS medium supplemented with either 100 mM NaCl or 200 mM mannitol. Germination rates of progenies from PVX-infected plants were higher than those of mock-inoculated progenies in both Wt and NahG genotypes (Figure S1). These findings suggest that responses elicited by SA-dependent signaling pathways are not likely involved in the enhanced tolerance to abiotic stress observed in progenies of PVX-infected *N. benthamiana* plants.

**Figure 3:**
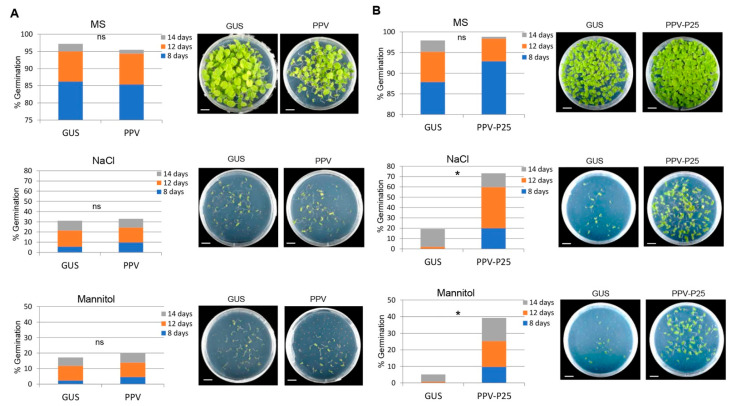
Effects of abiotic stress in the progeny of *N. benthamiana* plants infected with either PPV or PPV-P25: (**A**) germination rates of progenies derived from mock-inoculated plants (Gus) and plants infected with PPV on MS medium alone (upper panel) or supplemented with either 100 mM NaCl (middle panel) or 200 mM mannitol (lower panel); (**B**) germination rates of progenies derived from mock-inoculated plants (Gus) and plants infected with PPV-P25 on MS medium alone (upper panel) or supplemented with either 100 mM NaCl (middle panel) or 200 mM mannitol (lower panel). Representative examples of the different treatments photographed at 14 days after sowing are shown. The accumulative germination rates were calculated at 8, 12 and 14 days after sowing. Values were obtained from two independent experiments using six progenies per treatment. The significant differences are indicated by asterisk (Student’s t-test, * *p* < 0.05); ns, not significative; bars in each panel represent 1 cm.

In a previous work, it was determined that the P25 protein of PVX was the main pathogenicity determinant responsible for an enhanced drought-tolerant phenotype in PVX-infected *N. benthamiana* plants [18]. To assess whether P25 also contributes to the tolerant phenotype to abiotic stresses observed in the offspring of PVX-infected plants, seeds derived from mock-inoculated plants and from plants infected with either PPV or a genetically engineered PPV recombinant expressing P25 (PPV-P25) were germinated on MS medium alone or supplemented with either NaCl (100 mM) or mannitol (200 mM). There were no significant differences in the germination rates of the progenies derived from mock-inoculated and PPV-infected plants under osmotic and salt stresses (Figure 3A). However, germination rates of progenies from PPV-P25-infected plants were higher than those of mock-inoculated progenies in the presence of NaCl and mannitol (3.8- and 7.7-fold, respectively) 14 days after sowing (Figure 3B). Moreover, seedlings derived from PPV-P25-infected plants exhibited significantly enhanced tolerance to 200 mM mannitol compared with those of mock-inoculated plants as revealed by the quantification of their relative fresh weights and the length of their main roots (Figure S2).

To further investigate whether the P25 protein of PVX alone confers tolerance to abiotic stress, Wt seeds and seeds from *N. benthamiana* transgenic plants expressing the P25 protein of PVX [28] were germinated on MS medium alone or supplemented with NaCl (Figure S3). The germination rate of Wt seeds was approximately 50% and that of P25 transgenic seeds was approximately 75% in the presence of 100 mM NaCl at 14 days after sowing.

**Figure 4:**
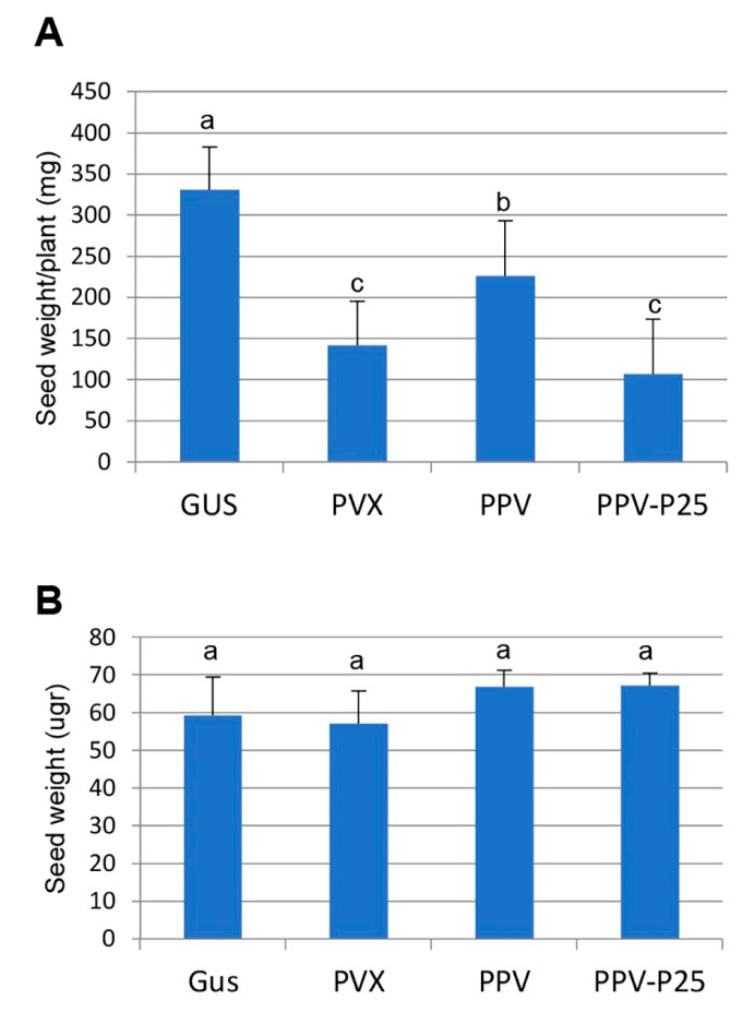
Effect of PVX, PPV and PPV-P25 infection on seed production and seed weight: (**A**) seeds from mock-inoculated (Gus) and virus-infected plants were weighted separately after threshing and recorded as seed weight per plant; (**B**) effect of virus infection on the weight of individual seeds. Seed weight was estimated after determining the weight of 80 seeds derived from each of eight plants per treatment. Statistically, comparisons between means were made among treatments by employing Duncan’s test. Different letters indicate significant differences between treatments.

The effect of virus infection on the reproductive fitness, i.e., virulence, of parental *N. benthamiana* plants infected with PVX, PPV and PPV-P25 was estimated by determining the weight of seeds per plant and compared with that produced by mock-inoculated plants. As shown in Figure 4A, plants infected with PPV showed a moderate reduction (1.5-fold) in seed production compared to mock-inoculated plants, whereas infection by PVX or PPV-P25 caused a more severe reduction in host fitness (2.3- and 3.1-fold, respectively). In addition, we observed that infection with the different viruses did not affect seed weight significantly (Figure 4B). Overall, these results indicate that infection by PVX and by the chimeric virus PPV-P25, but not by PPV, in parental *N. benthamiana* plants conferred tolerance to both salt and osmotic stresses in the next generation of plants, which correlated with the level of virulence of the pathogen.

### 2.2. Durability of the Transgenerational Tolerance to Abiotic Stresses

**Figure 5:**
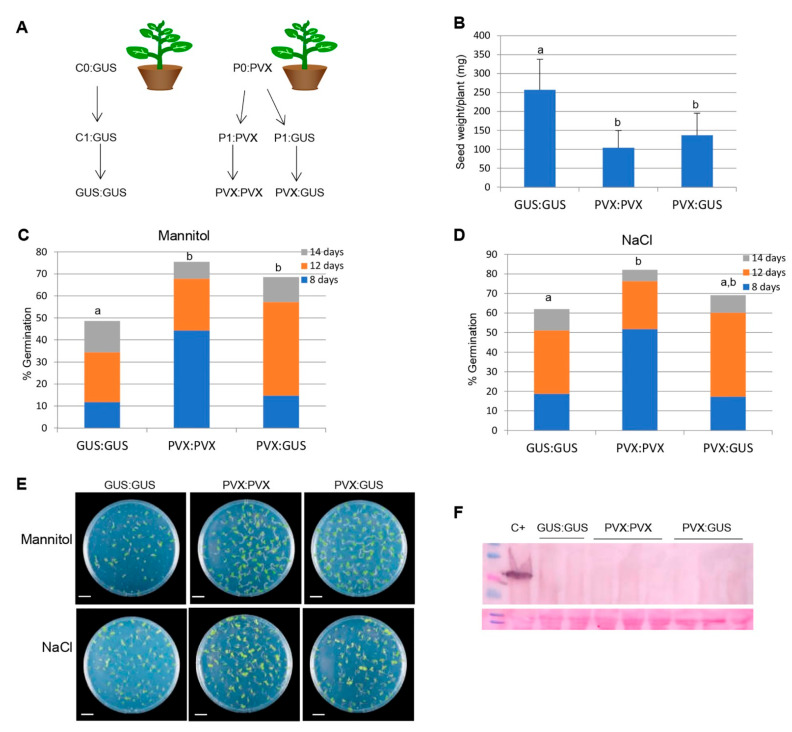
Transgenerational tolerance sustained over a virus-free generation: (**A**) experimental design for the generation of progeny lines. Parental *N. benthamiana* plants were mock-inoculated (C0:Gus) or inoculated with PVX (P0:PVX) after which plants were allowed to set seed to provide C1 and P1 progenies, respectively. C1 and P1 plants were allowed to set seed under virus-free conditions, providing GUS:GUS and PVX:GUS progenies, respectively. A separate batch of P1 plants was inoculated with PVX to provide PVX:PVX progeny; (**B**) seed production in C1 (GUS:GUS) and P1 (PVX:GUS, PVX:PVX) plants. Statistically, comparisons between means were made among treatments by employing Duncan´s test. Different letters indicate significant differences between treatments; (**C**,**D**) germination rates of GUS:GUS, PVX:GUS and PVX:PVX progenies on MS medium supplemented with either 200 mM mannitol (**C**) or 100 mM NaCl (**D**); (**E**) representative examples of the different treatments photographed at 14 days after sowing. The accumulative germination rates were calculated at 8, 12 and 14 days after sowing. Values were obtained from two independent experiments using six progenies per treatment; (**F**) Western blot analysis of seedlings extracts derived from C1 and P1 progenies using antibodies against PVX CP. The lower panel shows the Ponceau S-stained membrane after blotting as control of loading. Lane C+ corresponds to extract from a PVX-infected plant diluted 1:200. Bars in each panel represent 1 cm.

To examine the durability of the transgenerational tolerance to abiotic stresses, three individual plants from each of two different mock-inoculated and PVX-infected progeny lines were allowed to set seed under virus-free conditions, providing GUS:GUS and PVX:GUS progeny lines, respectively. In parallel, three individuals from each of two different PVX-infected progeny lines were infected with PVX to provide PVX:PVX progeny lines (Figure 5A). In two independent experiments, PVX:PVX progeny lines suffered severe fitness costs, as evidenced by a 2.5-fold reduction in seed production in comparison to mock-inoculated progeny lines (GUS:GUS) (Figure 5B). Remarkably, PVX:GUS treatment also had a detrimental effect on seed production (1.9-fold reduction) compared to GUS:GUS progeny lines. Nevertheless, GUS:GUS and PVX:GUS progeny lines did not differ in plant growth or seed weight (data not show). Germination rates of six independent PVX:PVX and PVX:GUS progeny lines were statistically higher compared with GUS:GUS in MS containing 200 mM mannitol (Figure 5C,E), whereas tolerance of PVX:GUS progeny lines was intermediate between that of PVX:PVX and GUS:GUS when seeds were germinated in MS with 100 mM NaCl (Figure 5D,E). We confirmed by Western blot analysis that seedlings derived from all the different second generation, progeny lines were not infected by PVX (Figure 5F). These results were repeated in several independent experiments; thus, it can be concluded that transgenerational tolerance to abiotic stresses is partially sustained over one virus-free generation, at the expense of reproductive fitness.

### 2.3. Role of RNA Silencing Pathways in Transgenerational Tolerance

**Figure 6:**
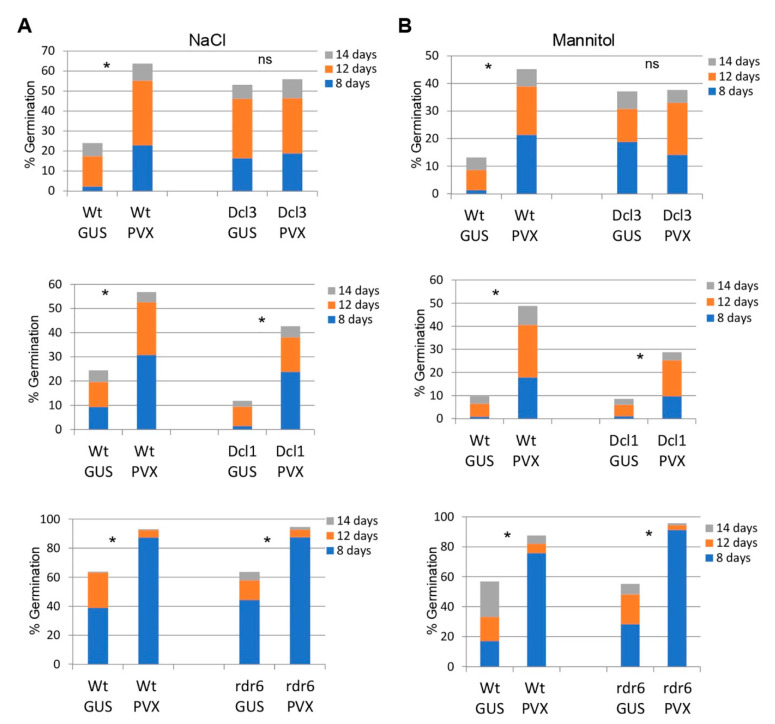
Tolerance to abiotic stress in the progeny of mutants of *N. benthamiana* impaired in RNA silencing pathways. Germination rates on MS medium supplemented with either 100 mM NaCl (**A**) or 200 mM mannitol (**B**) of progenies derived from mock-inoculated plants (Gus) and plants infected with PVX of transgenic lines suppressed for *Dcl3* (upper panels)*, Dcl1* (lower panels), *Rdr6* (lower panels) and wild-type (Wt) plants. The accumulative germination rates were calculated at 8, 12 and 14 days after sowing. Values were obtained from two independent experiments using six progenies per treatment. The significant differences are indicated by an asterisk (Student’s t-test, * *p* < 0.05); ns, not significative.

To examine the role of different RNA silencing pathways in virus-induced transgenerational tolerance to abiotic stresses, we compared transgenerational tolerance phenotypes between Wt plants and transgenic lines suppressed for *Dcl1*, *Dcl3* and RNA-directed RNA polymerase 6 (*Rdr6*). Seeds were collected from Wt and transgenic plants either infected with PVX or mock-inoculated and germinated on MS medium supplemented with either 100 mM NaCl or 200 mM mannitol (six independent progenies per treatment). The progenies derived from PVX-infected Dcl1 and Rdr6 lines expressed increased tolerance to both NaCl and mannitol in comparison to mock-inoculated Dcl1 and Rdr6 progenies. However, the progenies derived from the PVX-infected Dcl3 line failed to display increased tolerance to abiotic stresses in comparison to mock-inoculated Dcl3 progenies (Figure 6 and  Figure S4). Interestingly, progenies derived from Dcl3 plants either infected with PVX or mock-inoculated expressed significantly higher levels of tolerance to both NaCl and mannitol in comparison to mock-inoculated Wt progenies. These results suggest that Dcl3 mutant mimics the enhanced tolerance to abiotic stress observed in progenies of PVX-infected Wt plants.

### 2.4. Transcriptome Analysis of N. benthamiana Seeds

To study the transgenerational effects of PVX infection on the subsequent generation, we performed comparative RNAseq analyses on N. benthamiana seeds derived from parental plants of two different genotypes (Wt and Dcl3) that were either infected with PVX (WtPVX and Dcl3PVX) or mock-inoculated (Dcl3Ctr and WtCtr). Three biological replicates were analyzed for each treatment. Sequencing of 12 transcriptome libraries generated over 1151 million mapped reads. The proportion of total reads that mapped to the N. benthamiana genome of each sample was more than 95.28% and that of uniquely mapped reads was above 88.32%. More than 93.28% of the clean reads had quality scores at the Q30 level. The sequencing data are summarized in Table S1.

**Figure 7:**
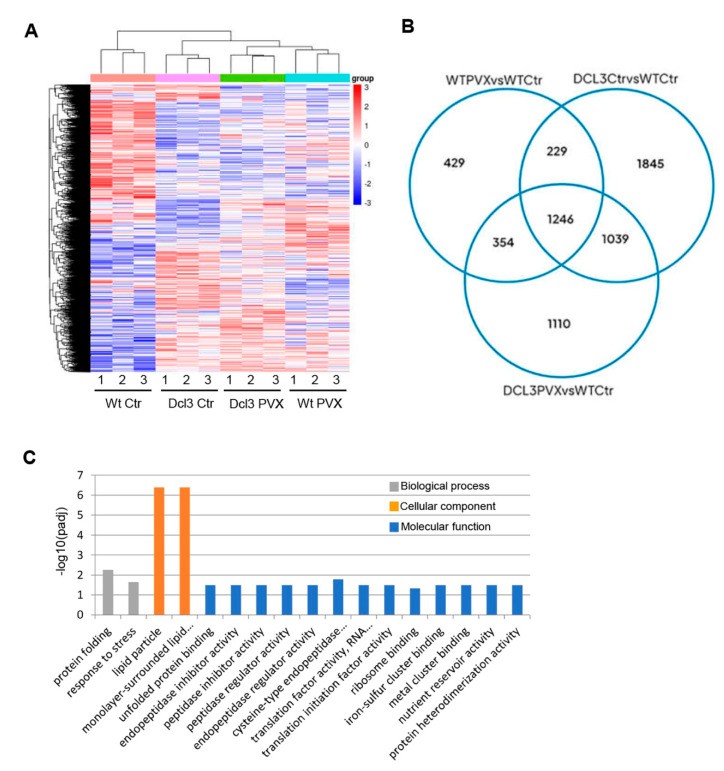
Transcriptomic profile of *N. benthamiana* seeds derived from Wt and Dcl3 parental plants either infected with PVX (WtPVX and Dcl3PVX) or mock-inoculated (Dcl3Ctr and WtCtr): (**A**) hierarchical cluster analysis of the expression profiles for the 7370 genes that were differential expressed (DEG) (*p* < 0.05) in at least one treatment. For each condition, the values of different biological replicates are shown. Expression values are colour-coded, with red indicating high expression levels, blue indicating low expression levels and white indicating no expression; (**B**) Venn diagrams displaying the number of specific and common DEGs found in the WtPVX vs. WtCtr, Dcl3Ctr vs. WtCtr and Dcl3PVX vs. WtCtr comparisons; (**C**) gene ontology (GO) analysis of DEGs in the WtPVX vs. WtCtr comparison. The x-axis represents the enriched GO term, and the y-axis represents the level of significance of GO enrichment, expressed as −log10(padj). Different colours are used to distinguish biological processes, cellular components and molecular functions.

An adjusted *p*-value < 0.05 was used to identify differentially expressed genes (DEGs) based on their normalized read counts. A total of 7370 DEGs were identified, and the mean abundance of genes ranged from 140.57 to 112.75 fragments per kilobase of transcript sequence per millions base pairs sequenced (FPKM) in the four treatments. Hierarchical clustering was used to group the entire DEG datasets from the four progenies by similarity of their overall gene expression profiles (Figure 7A). A separation of mock-inoculated Wt progenies (WtCtr) from the other progenies was evident, although Dcl3Ctr could also be distinguished from PVX-infected progenies (WtPVX and Dcl3PVX). This suggests that the suppression of Dcl3 functions produces significant transcriptomic changes in addition to those imposed by virus infection.

To study the effects of the parental stress/genotype treatment on the progenies, paired comparisons were made between treatments. In the WtPVX vs. WtCtr comparison, 2258 DEGs, including 1120 upregulated and 1138 downregulated genes, were identified (Figure 7B, Table S1), whereas 4359 (2003 upregulated and 2356 downregulated) and 3649 (1814 upregulated and 1835 downregulated) DEGs showed significant changes in the Dcl3Ctr vs. WtCtr and Dcl3PVX vs. WtCtr comparisons, respectively.

Gene ontology (GO), an internationally standardized gene function classification system, was used to classify the DEGs in each dataset (Table S2). DEGs can be categorized into three main categories: biological process, cellular component and molecular function. We focused on GO terms within biological processes that were overrepresented (hypergeometric test, *p* < 0.05) in the WtPVX vs. WtCtr comparison to highlight qualitative differences when compared to datasets from the rest of comparisons. GO enrichment analysis allowed the identification of only two biological processes, i.e., protein folding and response to stress, overrepresented in the WtPVX vs. WtCtr comparison (Figure 7C). It is noteworthy that, among others, the GO terms protein folding and response to stress were also overrepresented in the set of DEGs from Dcl3Ctr vs. WtCtr and Dcl3PVX vs. WtCtr comparisons, respectively (Table S2). Cellular component and molecular function categories related to lipid particle, unfolded protein binding, peptidase regulator activity, and translation initiation factor activity, among others, were also overrepresented in the WtPVX vs. WtCtr comparison.

The WtPVX vs. WtCtr comparison differentially altered the expression of 24 and 38 DEGs classified in protein folding and response to stress categories, respectively (Table S3). DEGs in these categories comprise pathogenesis- or stress-related proteins, including chaperone proteins, universal stress protein A (*UspA*), glutathione peroxidase1 (*Gpx1*) and late embryogenesis abundant protein5 (*Lea5*), among others. Many of the DEGs within the protein folding category were also categorized into the response to stress category. Among the 38 DEGs belonging to the response to stress category, 19 showed upregulation while the remaining 38 showed downregulation in the WtPVX vs. WtCtr comparison. A total of 9 DEGs classified in the protein folding category were induced, whereas 15 DEGs were downregulated. Several of these DEGs were also altered in the datasets from Dcl3Ctr vs. WtCtr and Dcl3PVX vs. WtCtr comparisons (Table S3).

### 2.5. Validation of RNAseq Analysis

**Figure 8:**
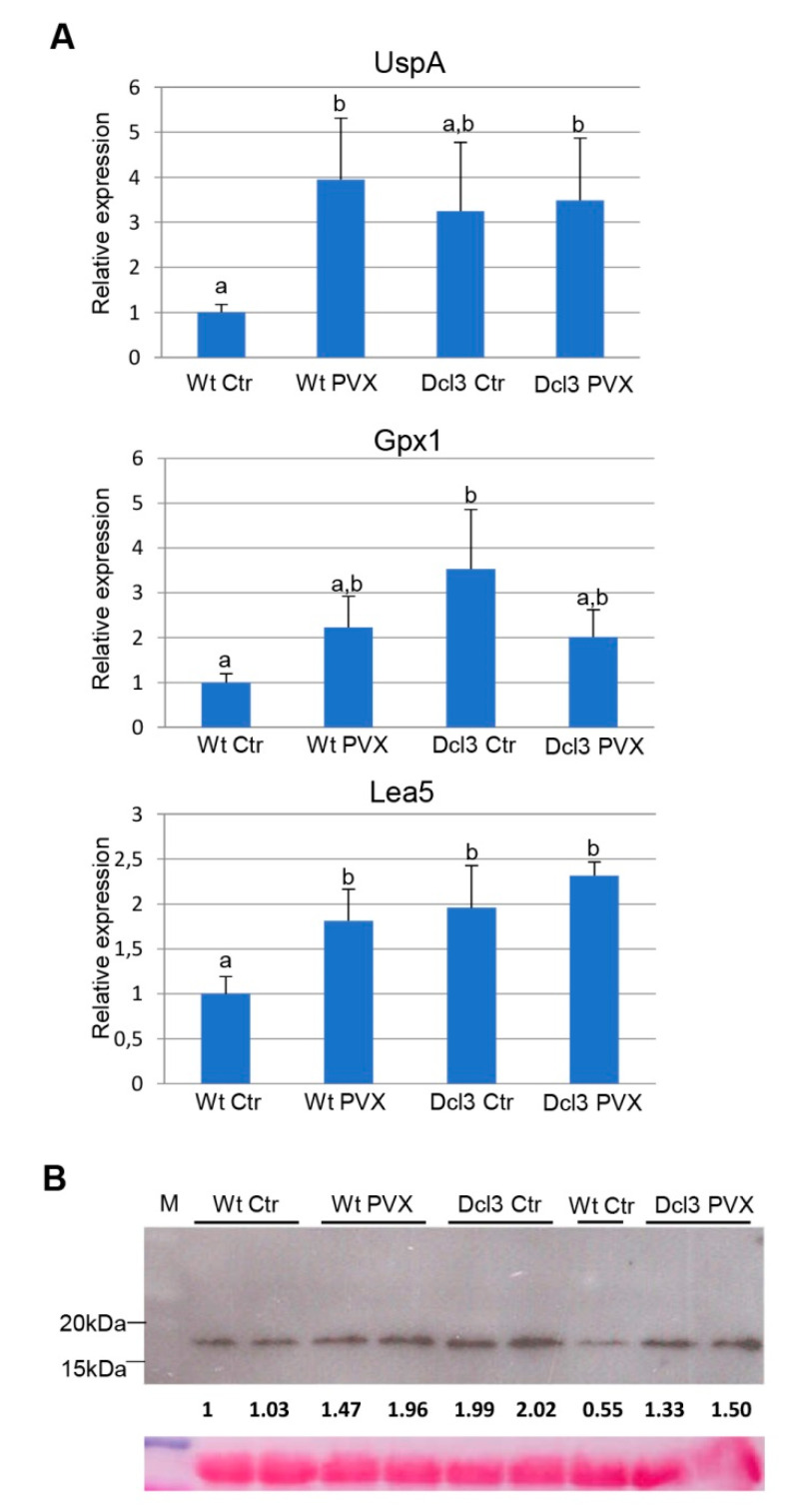
Validation of RNA-Seq data of representative genes from the gene ontology categories protein folding and response to stress: (**A**) the relative expression levels of universal stress protein A (*UspA*), glutathione peroxidase1 (*Gpx1*) and late embryogenesis abundant protein5 (*Lea5*) were determined in seed extracts derived from WtCtr, WtPVX, Dcl3Ctr and Dcl3PVX progenies by quantitative real-time PCR. Statistically, comparisons between means were made among treatments by employing Duncan’s test. Different letters indicate significant differences between treatments; (**B**) Western blot analysis of seed extracts derived from WtCtr, WtPVX, Dcl3Ctr and Dcl3PVX progenies using antibodies against sunflower CI sHSP. The lower panel shows the Ponceau S-stained membrane after blotting, as control of loading. The intensity of each CI sHSP band was quantified by densitometry analyses.

Differential expression of a subset of upregulated stress-associated genes (*Usp-A*, *Gpx1* and *Lea5*) was determined by quantitative real-time PCR (qRT-PCR) using RNA preparations extracted from a new set of seeds (not used for RNAseq) derived from WtCtr, WtPVX, Dcl3Ctr and Dcl3PVX (Figure 8A). These candidate genes were selected for their predicted biological functions associated with tolerance to stress, potentially contributing to the transgenerational effects. In general, the expression levels of *Usp-A*, *Gpx1* and *Lea5* measured by qRT-PCR in the different treatments confirmed the pattern observed by RNAseq analyses (Table S3). The relative accumulation of the mRNAs was greater in progenies derived from WtPVX compared to WtCtr, although the absolute values determined by qRT-PCR were higher than those derived from RNAseq data. In addition, progenies derived from Dcl3 plants either infected with PVX or mock-inoculated expressed significantly higher levels of *Usp-A*, *Gpx1* and *Lea5* mRNAs in comparison to mock-inoculated Wt progenies (WtCtr).

Having shown upregulation of several small chaperonins classified in the protein folding GO category within the WtPVX vs. WtCtr comparison (Table S3), we next sought to validate these findings by Western blot analysis. *CI sHSP* encodes a class of cytosol-localized small heat shock protein accumulating in mature seeds [29]. The availability of antibodies against sunflower CI sHSP allowed us to investigate the expression of this protein in extracts from *N. benthamiana* seeds derived from WtCtr, WtPVX, Dcl3Ctr and Dcl3PVX (Figure 8B). CI sHSP accumulated at higher levels in seeds derived from WtPVX, Dcl3Ctr and Dcl3PVX progenies compared to WtCtr (1.4- to 2-fold). The relative gene expression pattern was very similar to that observed by RNAseq, where increases ranged from 1.2 to 1.4 between different small chaperonins present in the WtPVX vs. WtCtr comparison.

## 3. Discussion

In this study, we report that in addition to bacteria, insect herbivory and chemical treatments [7,8,10], RNA viruses can also induce a transgenerational response in the form of tolerance to abiotic stresses in the progeny of virus-infected plants. Previously, it was reported that the progeny of plants infected with TMV exhibited a higher level of resistance to viral, bacterial and fungal infection as compared with the progeny of uninfected plants [9]. Our study expands previous findings in that plants primed with viruses produce progenies with an enhanced tolerance to abiotic stresses, i.e., salt and osmotic stress, at the expense of reproductive fitness.

### 3.1. P25 Protein of PVX Is A Major Contributor to Transgenerational Tolerance to Abiotic Stress

Tolerance to salt and osmotic stress measured as germination rates and seedling establishment was exhibited in the progeny of plants infected with PVX and the chimeric virus PPV-P25, but not with PPV. Thus, the P25 protein of PVX is a major pathogenicity determinant that contributes to the tolerance to abiotic stresses in the progenies of virus-infected plants. In a previous work, it was shown that expression of P25 by PPV led to an increase of PPV virulence and to a concomitant drought-tolerant phenotype in parental *N. benthamiana* plants [18,30]. The expression of the SA-responsive gene pathogenesis-related protein 1 (*PR-1*), as well as transcript levels of the defense-related genes *Hin1* and *Hsr203j,* were increased at much higher levels in PPV-P25-infected plants compared with plants infected with PPV [31]. In addition, metabolic and hormonal data further supported an altered physiological status in plants infected with PPV-P25 of more amplitude than the one triggered by PPV infection [18]. Moreover, transgenic expression of the PVX P25 protein alone had a deep impact on the transcriptome of tobacco plants, with the most significant groups of the upregulated genes being those associated with responses to biotic and abiotic stresses [32]. Indeed, we show here that P25 transgenic seeds were more tolerant to NaCl than Wt seeds. Thus, we propose that the P25 protein of PVX would induce metabolic adaptation in plants to abiotic stress as a result of its virulence properties and that this feature is transmitted to the next generation of plants. No data have been reported on seed transmission of PVX or PPV in *N. benthamiana*, ruling out the possibility that tolerance to abiotic stresses in the progeny was due to vertical transmission of the viruses from the infected mother plants to their progeny [26].

### 3.2. Virulent Virus Infection Induces Transgenerational Responses

Numerous studies have documented the relationship between the establishment of plant responses to biotic stresses in parental plants and tolerance to different pathogens in the following generations [7,8,9,10]. The cause for the increased tolerance to abiotic stresses in the progeny of plants infected with virus may be that plants use a network of interconnected signaling pathways to deal with various environmental stresses and that several of these responses are common in coping against biotic and abiotic stresses [33,34]. Indeed, plant responses to viruses, included PVX, involve metabolic, physiological and transcriptomic changes that overlap, at least partially, with the responses elicited by abiotic stresses [35,36]. We conjecture that virus infections that trigger defense responses beyond a threshold level in the parental plants would induce tolerance to abiotic stresses in the next generation of plants. Consequently, this transgenerational response to virus infection was correlated with an increased level of virulence, i.e., a more pronounced deleterious effect of the pathogen on the host reproductive fitness [17].

Several virus infections, including that caused by PVX, lead to an increase in the accumulation of several plant hormones, e.g., SA, JA and abscisic acid [16,37]. It has been reported that transgenerational resistance to *P. syringae* in Arabidopsis was promoted by the priming of SA-inducible defense genes [7]. Here, we showed that tolerance to abiotic stress in the progeny of PVX-infected plants was not compromised in plants defective in SA accumulation (NahG line). Accordingly, our results suggest that transgenerational responses induced by virus infection are largely independent of SA accumulation and that additional hormonal signaling cascades contribute to this phenotype.

RNAseq analysis shown in this work support an ample transcriptomic reprogramming in progenies derived from PVX-infected Wt plants compared to mock-inoculated plants, which could be responsible, at least in part, for the tolerance to abiotic stress. The induced expression of a subset of genes classified in GO in terms of protein folding and response to stress (chaperone proteins, *UspA*, *Gpx1* and *Lea5*, among others), suggest that the progenies derived from PVX-infected plants were primed for enhanced tolerance against abiotic stresses. It has been reported that overexpression of the *Usp* from *Salicornia brachiata* mitigates salt and osmotic stress in transgenic tobacco plants [38]. The overexpression of *OsLea5* in rice plants enhanced the tolerance to drought and salt stress by the activation of ABA-mediated antioxidant defenses [39]. Expression studies on different crop species have shown pronounced induction of several *Gpx* genes in response to abiotic and biotic stress factors, which suggest important roles of Gpxs in stress defense and tolerance [40]. In addition, OsHSP18.2, a small cytosolic HSP from rice, has been implicated in seed vigor and improved germination and seedling establishment under abiotic stresses by reducing deleterious accumulation of reactive oxygen species in seeds [41]. Overall, these findings argue for an association between altered expression of these stress-related genes and the enhanced tolerance to abiotic stress observed in progenies of virus-infected plants.

### 3.3. Persistence of Transgenerational Tolerance over Time

Several studies have documented that transgenerational priming can be maintained over one stress-free generation [7,10]. Our study has not formally demonstrated whether the inheritance of tolerance to abiotic stress in progenies derived from virus-infected plants is a maternal effect transmitted through the seed, for instance via the storage of stress-related proteins or defensive metabolites, or a longer term effect that is inherited in an epigenetic manner. However, although to a lesser extent than in progeny of virus-infected plants, tolerance to abiotic stress was even sustained over one virus-free generation, suggesting that the phenomenon is epigenetically regulated. Moreover, in contrast to Wt, dcl1 and rdr6 progenies, progenies from PVX- and mock-inoculated Dcl3 plants showed no difference in tolerance to salt and osmotic stress, while the mock-inoculated progeny of this mutant showed constitutively increased tolerance to abiotic stress in comparison to progeny from mock-inoculated Wt plants. Progenies from mutants of the RdDM pathway, e.g., *ago4*, *drm1drm2cmt3*, and *drm1drm2kyp*, have been reported to express constitutively elevated levels of resistance to *H. arabidopsidis* in comparison to progenies of Wt plants [7,42]. Furthermore, the overall pattern of gene expression, including genes in the GO categories protein folding and response to stress, in progenies derived from PVX- and mock-inoculated Dcl3 plants were grouped together with that of Wt plants infected with PVX by hierarchical clustering, indicating a closer relationship in the changes of transcript abundance compared to those altered in the mock-inoculated Wt progeny. *DCL3* encodes a homolog of Dicer involved in small RNA pathways implicated in epigenetic regulation [14]. Several works have suggested the involvement of siRNA biogenesis pathways in the establishment of transgenerational responses to environmental stresses mediated by RdDM [10,42,43]. Since the Dcl3 mutant is affected in RdDM, our results support a role of siRNAs, and thereby DNA methylation, during the onset of transgenerational tolerance to abiotic stresses in progenies of virus-infected plants. In this sense, it has been shown that virus infection causes changes in the expression of transcriptional gene silencing factors with RdDM activities, particularly repression of genes encoding DCL3 and the RdDM effectors Argonaute9 (AGO9) and AGO4, which correlate with changes in methylation at the whole genome level [44]. It is tempting to speculate that infection by PVX leads to misregulation of several components of the RdDM pathway that would repress DNA methylation, thereby producing changes in inducibility of stress-related genes in the progeny of infected plants.

### 3.4. Reproductive Costs Associated with Transgenerational Responses

Previously, it was shown that the benefits of priming in BABA-treated parental plants outweighed the reproductive costs caused by infection with *P. syringae* or *H. parasitica* [15]. However, reproductive fitness associated with transgenerational responses in the progeny of plants primed with a pathogen has not been extensively explored. López Sánchez et al. [5] did not observe consistent effects on plant growth or seed set in F1 and F2 progeny of plants primed with a biotrophic or a necrotrophic pathogen. Here, we show that transgenerational responses induced by virus infection, albeit sustained over one virus-free generation, had a detrimental effect on seed production in the offspring of infected plants compared to control parents. These results suggest that the improved tolerance to abiotic stresses in the progeny of virus-infected plants is potentially traded-off with another important life history trait, such as fecundity. Similar findings have been reported in *Manduca sexta* treated with the bacterial priming agent peptidoglycan, where the benefits which the offspring gains from transgenerational responses are countered by reduced reproductive power [45]. Thus, from an environmental point of view, the significance of virus-induced transgenerational tolerance to abiotic stress could be questionable, as its induction was offset by major reproductive costs arising from a detrimental effect on seed production. Reduced seed set could reflect a reallocation of resources from seed production to plant defense in the progeny of virus-infected plants. In plants exposed to abiotic stress conditions that lead to a severe reduction in fitness, tolerance to abiotic stress in the progeny of virus-infected plants could compensate for detrimental effects on reproductive performance.

In conclusion, it has been previously reported that infection of plants by different microorganisms rendered progenies with increased tolerance to biotic stresses. Here, we expand previous findings to show that infection by virulent viruses induces transgenerational tolerance to osmotic and salt stress, which was partially sustained over one stress-free generation at the expense of reproductive fitness. Furthermore, the Dcl3 mutant that is affected in RdDM mimicked the transgenerational phenotype of tolerance to abiotic stress observed in progenies of virus-infected Wt plants, suggesting that small RNA pathways provide the machinery for transgenerational responses. Although the range of virus–host combinations in this study is limited, our findings would lead to the hypothesis that progenies of plants infected with viruses will experience increased tolerance to environmental stresses compared to progenies of non-infected plants under some of the projected climate change scenarios. Altogether, the work presented could open the way for future studies deciphering how plants modulate responses to virus diseases towards transgenerational tolerance against recurrent environmental stresses such as salt and drought.

## 4. Materials and Methods

### 4.1. Plant Material

The N. benthamiana transgenic plants expressing the salicylate hydroxylase gene and the P25 protein of PVX have been described previously [28,46]. N. benthamiana plants expressing hairpins that target endogenous DCL1, DCL3 and RDR6 transcripts were described by Dadami et al. [47] and Schwach et al. [48], respectively. Plants were kept in environment-controlled growth chambers with 16/8 h day/night photoperiod, ~2500 lux of daylight intensity and 60% relative humidity.

### 4.2. Binary Vector Constructs and Agro-Inoculation

The binary vector pGR107 expressing the infectious cDNA of PVX has been previously described [48]. pGWBinPPV-3xHA is a derivative of pBinPPV which harbors the infectious cDNA of PPV [49]. PPV expressing PVX P25 (PPV-P25) sequences was described before [30]. pCAMBIA1305.1 containing a β-glucuronidase (*GUS*) gene was used as negative control. Four-week-old plants were agro-infiltrated with *Agrobacterium tumefaciens* bearing the indicated binary vectors [50]. Plants were grown at 25 °C using a 16/8 h day/night photoperiod. All parental plants were allowed to set seed under similar growth conditions. Seeds were weighted separately after threshing from 8 plants per treatment and recorded as seed grain per plant. Seed weight was estimated after determining the weight of 80 seeds derived from each of four treatments.

### 4.3. Stress Treatments

*N. benthamiana* seeds were surface sterilized in 70% ethanol for 90 s, followed by 10% household bleach solution (3.7% active chlorine) for 12 min, then washed five times with sterile distilled water. The seeds were placed in MS medium with 0.7% sucrose and solidified with 0.8% (*w*/*v*) agar alone or supplemented with either 100 mM NaCl or 200 mM mannitol. Germination rates were calculated from the percentage of seeds with radicles protruding through the seed coat. The assays were performed with approximately 100 seeds per progeny, using six progenies per treatment. Seeds were geminated in the growth chamber with a 16/8 h day/night photoperiod at 25°C. For the rest of the experiments, osmotic and salt stress treatments were performed by transferring seedlings grown in Petri dishes under standard conditions to plates containing MS medium alone or supplemented with either 200 mM mannitol or 150 mM NaCl for 13 days. Whole tissues of both control and stressed seedlings were harvested, and fresh weights and the length of main roots were recorded. Values of seedlings grown on mannitol or NaCl were represented relative to the corresponding values of seedlings grown on MS medium.

### 4.4. Library Preparation for Transcriptome Sequencing

Total RNA was extracted from *N. benthamiana* seeds (20 mgr) using the procedure described by Suzuki et al. (2004) [51]. Three independent biological replicates were used to monitor differences in gene expression between treatments. The integrity and quality of the total RNA were checked using NanoDrop 2000 Spectrophotometer (Thermo Scientific, Waltham, MA, USA) and formaldehyde agarose gel electrophoresis. The transcriptome libraries, sequencing and bioinformatics analysis were performed at Novogene in United Kingdom; mRNA was purified from total RNA using poly-T oligo-attached magnetic beads. After fragmentation, the first strand cDNA was synthesized using random hexamer primers, followed by the second strand cDNA synthesis. The library was checked with Qubit and real-time PCR (Rotor-Gene Q thermal cycler, Qiagen, Hilden, Germany) for quantification and bioanalyzer for size distribution detection. Quantified libraries were pooled and sequenced on Illumina NovaSeq PE150 Platform according to effective library concentration and data amount. The clustering of the index-coded samples was performed according to the manufacturer’s instructions. After cluster generation, the library preparations were sequenced and paired-end reads were generated.

Raw reads were firstly processed through in-house Perl scripts. In this step, clean reads were obtained by removing reads containing adapter, reads containing ploy-N and low quality reads from raw data. At the same time, Q30 and GC content of the clean data were calculated. All the downstream analyses were based on the clean data with high quality.

The *N. benthamiana* reference genome and gene model annotation files were downloaded from the SGN ftp site (ftp://ftp.solgenomics.net/genomes/Nicotiana_benthamiana/assemblies (accessed on 16 October 2022)) directly. An index of the reference genome was built using Hisat2 v2.0.5 and paired-end clean reads were aligned to the reference genome using Hisat2 v2.0.5. FeatureCounts v1.5.0-p3 was used to count the reads numbers mapped to each gene. The expected number of FPKM of each gene was calculated based on the length of the gene and reads count mapped to this gene.

### 4.5. GO Enrichment Analysis of Differentially Expressed Genes

Differential expression analysis of the two assayed treatments (three replicates per treatment) was performed using the DESeq2 R package (1.20.0) [52]. The resulting *p*-values were adjusted using the Benjamini and Hochberg’s correction for controlling the false discovery rate. Genes with an adjusted *p*-value < 0.05 were assigned as differentially expressed. Fold-change calculations were performed for paired-comparisons made between treatments.

Gene ontology (GO) enrichment analysis of differentially expressed genes was implemented by the clusterProfiler R package (v3.8.1), in which gene length bias was corrected. GO terms with adjusted *p* value less than 0.05 were considered significantly enriched by the DEGs.

### 4.6. qRT-PCR and Protein Gel Blot Analysis

Total RNA from seeds was prepared as indicated above; qRT-PCR for the analysis of gene expression was performed with gene-specific primers (Table S3). The relative quantification of PCR products was calculated by the comparative cycle threshold (ΔΔCt) method as described [53]. Amplification of 18S rRNA was chosen for normalization because of its similar level of expression across all treatments. All qRT-PCR experiments were performed in triplicate.

For the detection of PVX, total proteins were extracted by grounding leaf disks with a pestle and mixed with five volumes of extraction buffer (0.1 M Tris-HCl PH 8, 10 mM EDTA, 0.1 M LiCl, 1% β-mercaptoethanol and 1% SDS) [54]. Samples were boiled and fractionated in 15% SDS-PAGE gels. A commercial rabbit antibody (1:1000 dilution) was used (No. 070375/500; Loewe Biochemica GmbH, Germany).

Tobacco seeds (ca 5 mgr) were frozen in liquid nitrogen and ground to a powder. The homogenate was resuspended using the extraction buffer: 8 M urea, 40 mM Tris-HCl, 20 mM DTT, 2% Tween-20 and 5 mM PMSF at a 1/20 (*w*/*v*) seed weight/buffer ratio and incubated for 1 h at 4 °C, vortexing every 10 min to facilitate protein extraction. The homogenates were subsequently centrifuged at 15 °C for 30 min at 18,000 g. Aliquots of crude extracts were protein assayed (Bradford) using BSA as a standard protein. For detection of CI sHSP, a homemade antibody (1:700 dilution) was used as previously described [55]. Blotted proteins were detected using commercial secondary antibodies and Sigma Fast^TM^BICP/NBT substrate tablets (SIGMA Aldrich, Saint Louis, MI, USA). Densitometric analysis of blotted protein bands was performed using the public domain software ImageJ (v1.52p) (National Institutes of Health website, image processing and analysis in java).

### 4.7. Statistical Analysis

All statistical analyses were performed using the statistical software IBM SPSS Statistics v.25 (IBM Corp). For each experiment, samples were assessed for normality via the Shapiro–Wilk test and for equality of variances using Levene’s test. For experiments with normally distributed samples of equal variance, one-way ANOVA followed by Duncan’s post, hoc test was performed. Otherwise, a nonparametric Mann–Whitney U test was employed, with the Bonferroni correction for multiple comparisons between samples applied. For comparisons between pairs of means (pairwise comparisons)*,* Student’s *t*-tests or Mann–Whitney U tests were employed, depending on the normality of data.

## Data Availability

RNA-seq data were uploaded to the Gene Expression Omnibus (GEO, http://www.ncbi.nlm.nih.gov/geo (accessed on 16 October 2022)). The accession number is GSE214636.

## Supplementary Materials

The following supporting information can be downloaded at: https://www.mdpi.com/article/10.3390/ijms232012497/s1.
