# Supplementary figures and images for: Transgenerational Tolerance to Salt and Osmotic Stresses Induced by Plant Virus Infection
Source: Int J Mol Sci. 2022 Oct 18;23(20):12497. doi: 10.3390/ijms232012497 (PMC9604408; doi:10.3390/ijms232012497)

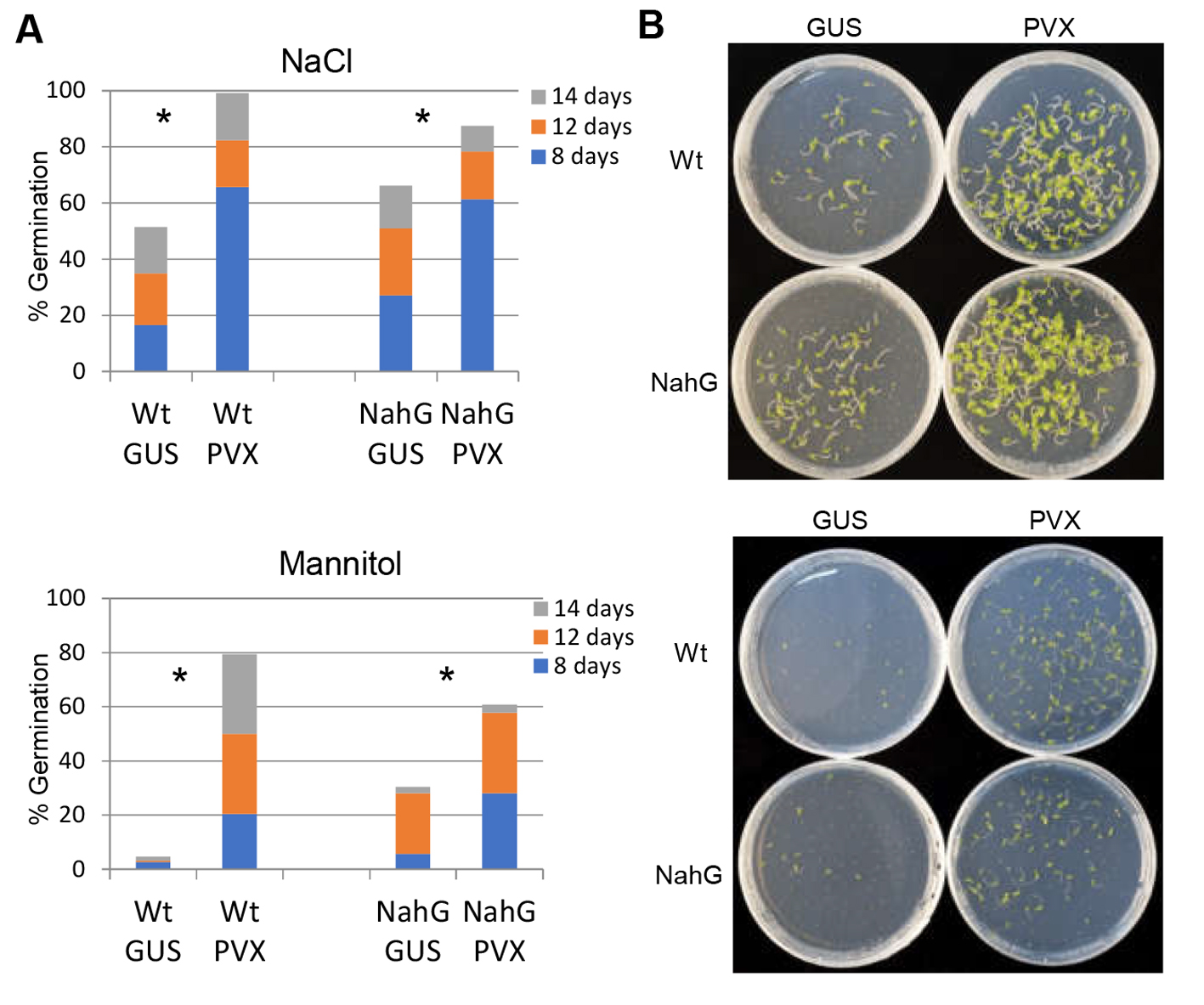

Supplement: Supplementary file 1 [file ijms-23-12497-s001.zip › figs S1. Tolerance to abiotic stress in the progeny of PVX-infected, salicylic acid- (SA) deficient N. ben-thamiana plants.jpg]

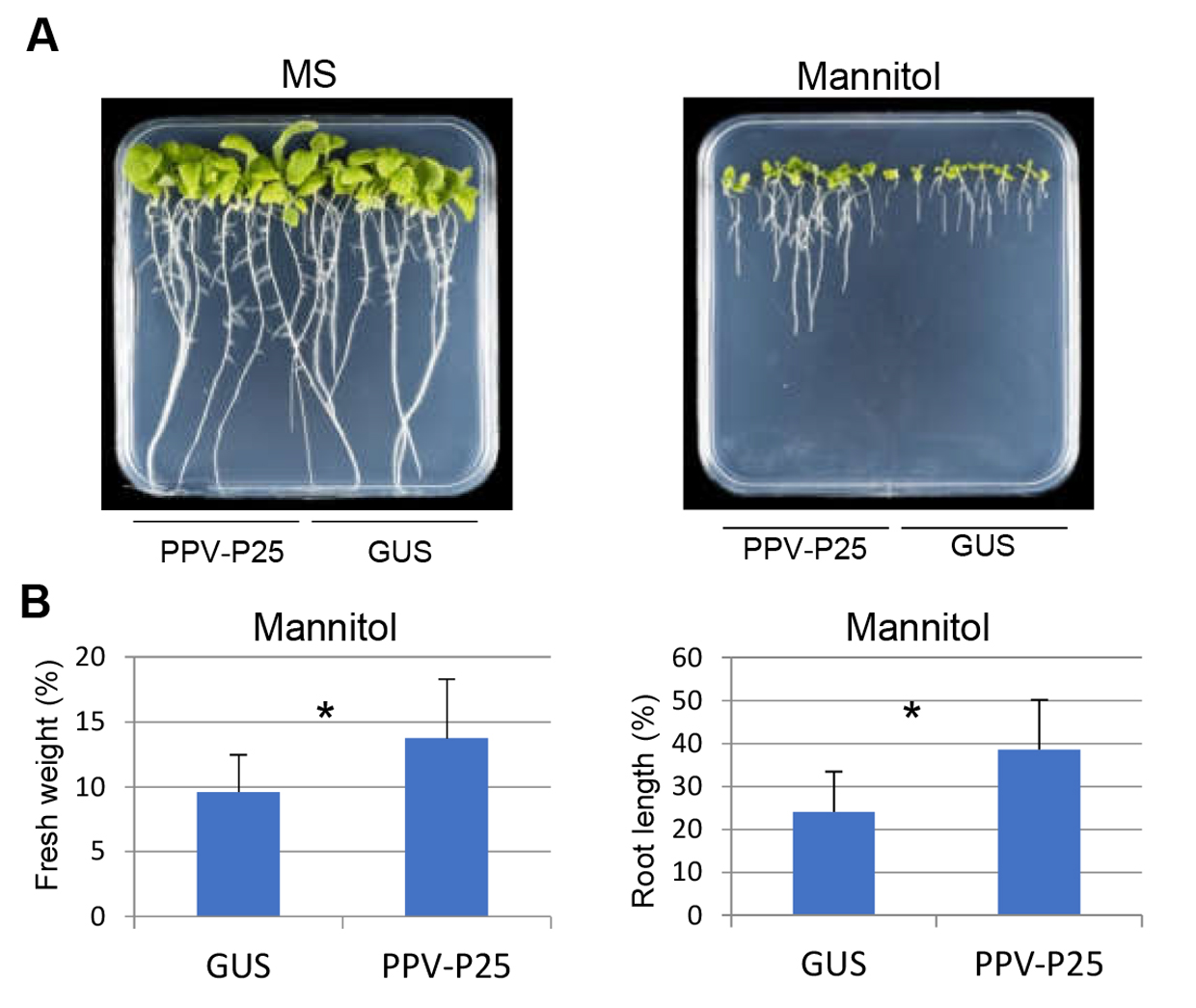

Supplement: Supplementary file 1 [file ijms-23-12497-s001.zip › figs S2. Tolerance to abiotic stress on the growth of progeny seedlings derived from PPV-P25-infected N. benthamiana plants.jpg]

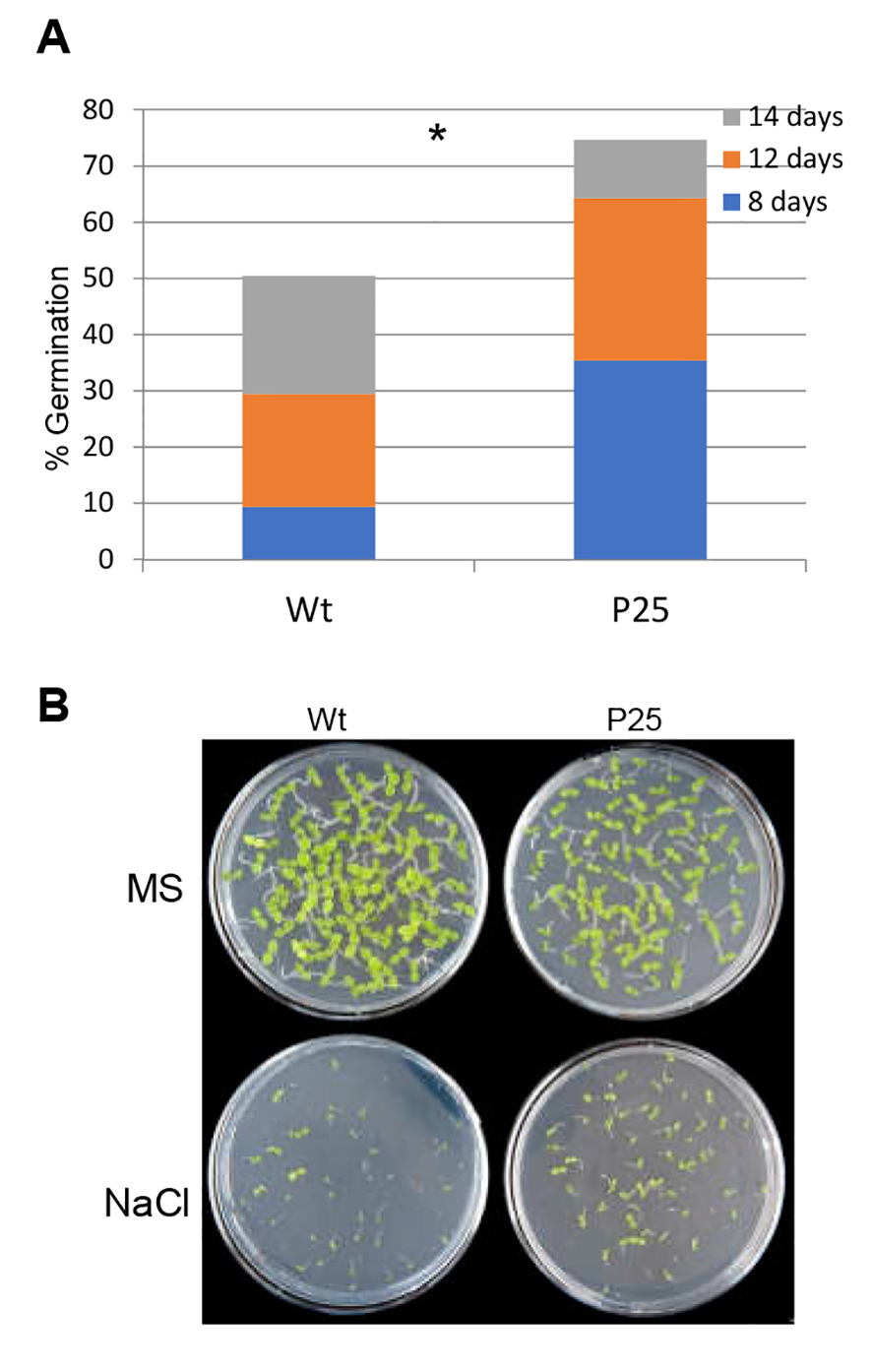

Supplement: Supplementary file 1 [file ijms-23-12497-s001.zip › figs S3. Tolerance to abiotic stress in N. benthamiana transgenic plants expressing the P25 protein of PVX.jpg]

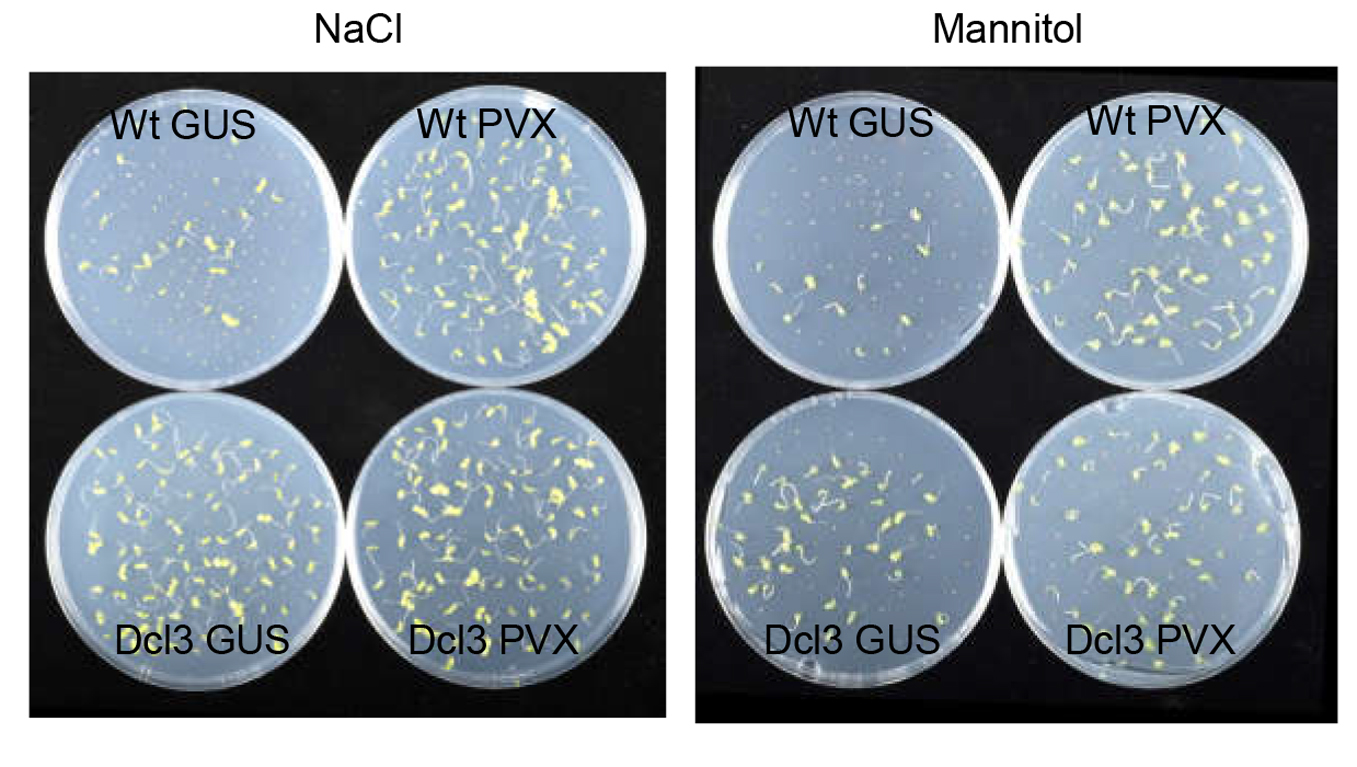

Supplement: Supplementary file 1 [file ijms-23-12497-s001.zip › figs S4. Tolerance to abiotic stress in Dcl3 mutant.jpg]
